# Pesticide residue detection techniques for increasing productivity and yield: recent progress and future outlooks

**DOI:** 10.3389/fpls.2025.1694779

**Published:** 2025-11-17

**Authors:** Muhammad Umer, Abid Naseer, Mustansar Mubeen, Yasir Iftikhar, Rafia Umer, Ayesha Akram, Muhammad Tanveer Altaf, Essam H. Ibrahim, Ahmed Ezzat Ahmed, Mingzheng Duan

**Affiliations:** 1 College of Agronomy and Life Sciences, Zhaotong University, Zhaotong, China; 2 School of Breeding and Multiplication (Sanya Institute of Breeding and Multiplication), College of Tropical Agriculture and Forestry, Hainan University, Sanya, China; 3 Centre of Excellence for Citrus, College of Agriculture, University of Sargodha, Sargodha, Pakistan; 4 Department of Plant Pathology, College of Agriculture, University of Sargodha, Sargodha, Pakistan; 5 Department of Field Crops, Faculty of Agriculture, Recep Tayyip Erdoğan University, Rize, Türkiye; 6 Department of Biology, College of Science, King Khalid University, Abha, Saudi Arabia; 7 Blood Products Quality Control and Research Department, National Organization for Research and Control of Biologicals, Cairo, Egypt; 8 Prince Sultan Bin Abdelaziz for Environmental Research and Natural Resources Sustainability Center, King Khalid University, Abha, Saudi Arabia

**Keywords:** pesticide residue, MRLs, metabolites, plant health, green chemistry, biosensors, machine learning

## Abstract

The intensive use of pesticides in modern agriculture has significantly improved crop yield and food security but introduced serious health concerns due to the accumulation of pesticide residues in fruits and vegetables and the environment, posing serious health risks. This review comprehensively explores the various residue detection techniques and plant metabolomics as an emerging tool to unravel the biochemical and physiological consequences of pesticide exposure. The article critically evaluates current methodologies for pesticide residue analysis, encompassing sampling strategies, storage considerations, and a wide range of extraction techniques, including QuEChERS, solid-phase extraction (SPE), and emerging green alternatives such as supercritical fluid extraction and ultrasound-assisted extraction. A detailed comparison of analytical techniques particularly gas chromatography (GC), liquid chromatography (LC), mass spectrometry (MS), and novel non-separative methods such as biosensors and spectroscopy is presented, emphasizing sensitivity, specificity, and adaptability to complex matrices. Furthermore, the integration of metabolomics with advanced platforms such as machine learning, green chemistry principles, and microfluidic innovations is discussed as a transformative direction for future pesticide residue monitoring. The review is a novel compilation of conventional residue detection methods and emerging omics-driven, artificial intelligence (AI)-assisted approaches and identifies current limitations, including matrix interferences and regulatory disparities, and advocates for the harmonization of residue standards, alongside the development of cost-effective, high-throughput analytical platforms to ensure food safety, improve risk assessment, and enhance understanding of plant metabolic responses under pesticide stress. Moreover, multi-omics approaches can be more reliable in evaluating the quality of claimed organic farming products.

## 1 Introduction

**Figure 1:**
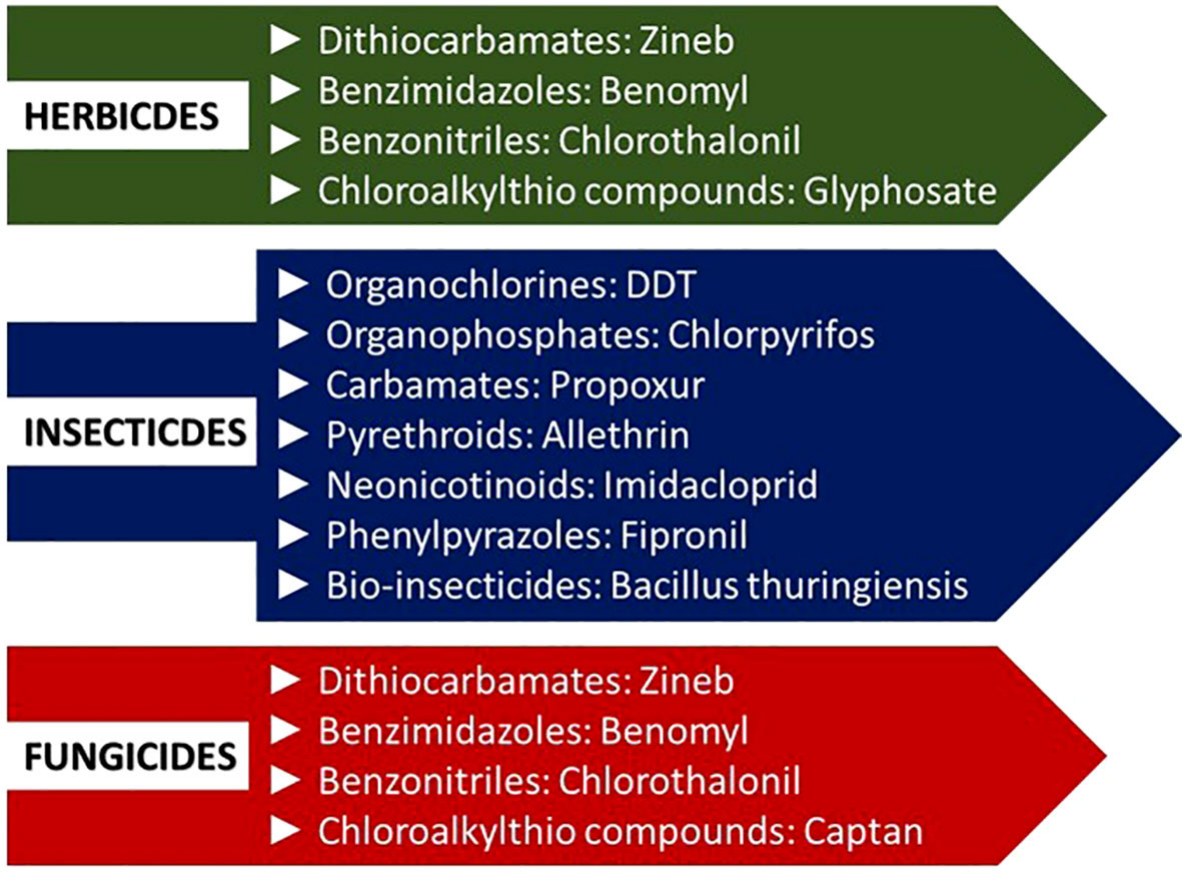
Schematic description of various pesticide classes.

Pesticides are chemical substances that control or eliminate pests, such as insects, rodents, fungi, weeds, and other unwanted organisms (Ahmad et al., 2024). These are categorized by their mode of action, chemical structure, risks, and use (Ahamad and Kumar, 2023; Khan et al., 2023). Pesticides can be categorized as i) organic pesticides, such as pyrethrins derived from flowers of chrysanthemum and neem oil; ii) inorganic pesticides, for example, copper sulfate used as a fungicide; iii) synthetic pesticides, such as organochlorines, organophosphates, carbamates, and synthetic pyrethroids; and iv) biopesticides derived from animals, plants, or microbes (Ahmad et al., 2024). On a broader level, pesticides can also be classified as insecticides, herbicides, and fungicides (**Figure 1**) based on their target pest. Globally, pesticides are sprayed in enormous volumes annually, forming a multi-billion dollar industry (González et al., 2021; Junaid and Gokce, 2024; Maino et al., 2023).

Generally, pesticides are applied by an aerial spray while dissolved in water or through granular application. Pesticides in granular form are spread over the soil surface, later dissolving and becoming available for action. Fogging is also the method of creating a fog of pesticide droplets that can penetrate dense foliage, targeting pests hiding in hard-to-reach areas (Abdulra’uf and Tan, 2012; Anastassiades et al., 2017). Pesticides are applied to seeds before planting to protect against soil-borne pests and diseases. A major amount of the applied pesticides is ultimately released into the environment, often contaminating the soil. Pesticide residues are detected in soil, groundwater, and surface water, where they may accumulate before being degraded and occasionally converted to even more toxic chemicals (Sabzevari and Hofman, 2022; Grondona et al., 2023). Microbial degradation is the powerful force that drives the transformation of pesticides in soils (Fernández-Alba et al., 2015; Hamilton et al., 2017). Certain pesticide degradation, primarily through hydrolysis, occurs via both biotic and abiotic pathways (Guerrero Ramírez et al., 2023). Photodegradation involves the breakdown of chemical bonds in insecticide molecules due to sunlight (El-Saeid et al., 2022). Some of the pesticides applied may stay on the surfaces of the treated plants or be moved to other parts of the plant. Extensively used pesticides can accumulate in plants, creating residues in agricultural products and finally making their way to the consumer (Rein et al., 2025). The movement and fate of applied pesticides are intricate, involving several processes: plants absorb pesticides from the soil through the xylem, take in substances from treated surfaces and the air through the cuticle and stomata, and transport them within the plant through both xylem and phloem. Additionally, pesticides can dissipate from treated surfaces and undergo biodegradation (Leskovac and Petrović, 2023; Swathy et al., 2024). The extent to which pesticides migrate from treated soil to various environmental compartments is determined by their chemical properties, soil characteristics, hydraulic loading, and agricultural management practices (Nuruzzaman et al., 2025). Some degraded residues and persistent pesticides are particularly concerning, as they can return to humans through bioaccumulation and biomagnification (Mitra et al., 2024). Plants metabolize pesticides through enzymatic pathways, transforming parent compounds into conjugated residues (Nicolopoulou-Stamati et al., 2016; Wang et al., 2014; Wong et al., 2013). Key enzymes include cytochrome P450s, glycosyltransferases, and ATP-binding cassette (ABC) transporters, which influence both the persistence and detectability of residues (Mao et al., 2020; Xie et al., 2021).

## 2 Pesticide residues affecting key metabolic pathways and plant health

The plant metabolism of pesticides involves a series of enzymatic reactions, primarily oxidation, reduction, hydrolysis, and conjugation, that transform pesticides into more water-soluble and less toxic metabolites. Key enzymes include laccases, glycosyltransferases, methyltransferases, and ABC transporters, which facilitate diverse metabolic pathways such as *S*-conjugate formation and glycosylation. These processes are regulated by plant hormones (e.g., salicylic acid, jasmonic acid, and brassinosteroids) and can be influenced by epigenetic mechanisms like DNA methylation and histone modification (Eerd et al., 2003; Zhang and Yang, 2021; Pszczolińska et al., 2024). The metabolic transformation of pesticides in plants is tissue- and development stage-specific, leading to variable residue profiles in leaves, flowers, and fruits. For example, in tomatoes, the parent compound cyantraniliprole remains the dominant residue, while its metabolites are present at much lower levels, with their abundance and type varying by tissue and maturity. Some metabolites may retain biological activity and contribute to overall toxicity, highlighting the importance of comprehensive residue analysis for risk assessment (Huynh et al., 2021; Pszczolińska et al., 2024).

### 2.1 Paths to metabolite alteration

Pesticide residues in plants lead to both direct and indirect changes in metabolite profiles.

#### 2.1.1 Enzyme activity modulation

Pesticide stress increases activities of detoxification enzymes (e.g., peroxidase and glutathione *S*-transferase), which in turn affect metabolic pathways and the abundance of specific metabolites (Zhang and Yang, 2021; Zhang et al., 2024a, 2024b).

#### 2.1.2 Metabolic pathway disruption

Residues can downregulate amino acids and phenolic compounds while upregulating flavonoids, impacting pathways such as amide biosynthesis and arginine/proline metabolism (Aprotosoaie, 2012; Zhang et al., 2024a).

#### 2.1.3 Formation of new metabolites

Plants metabolize pesticides into various transformation products, some of which may be more or less toxic than the parent compound. These metabolites can accumulate in different tissues and persist through developmental stages (Huynh et al., 2021; Kalyabina et al., 2021; Schusterova et al., 2021; Li and Fantke, 2023).

### 2.2 Types of metabolites affected

#### 2.2.1 Primary metabolites

Amino acids, organic acids, and sugars may be reduced or altered, affecting plant growth and stress responses (Zhang and Yang, 2021; Zhang et al., 2024b).

#### 2.2.2 Secondary metabolites

Flavonoids, phenolic acids, alkaloids, and terpenoids are often modulated, which can influence plant defense, nutritional quality, and aroma profiles (Zhang et al., 2024a, 2024b).

#### 2.2.3 Pesticide-derived metabolites

Plants generate a range of pesticide metabolites (e.g., glycosylated and hydroxylated forms) that can be detected in edible tissues and processed products like wine (Huynh et al., 2021; Kalyabina et al., 2021; Schusterova et al., 2021).

### 2.3 Plants’ metabolic response to pesticides

**Figure 2:**
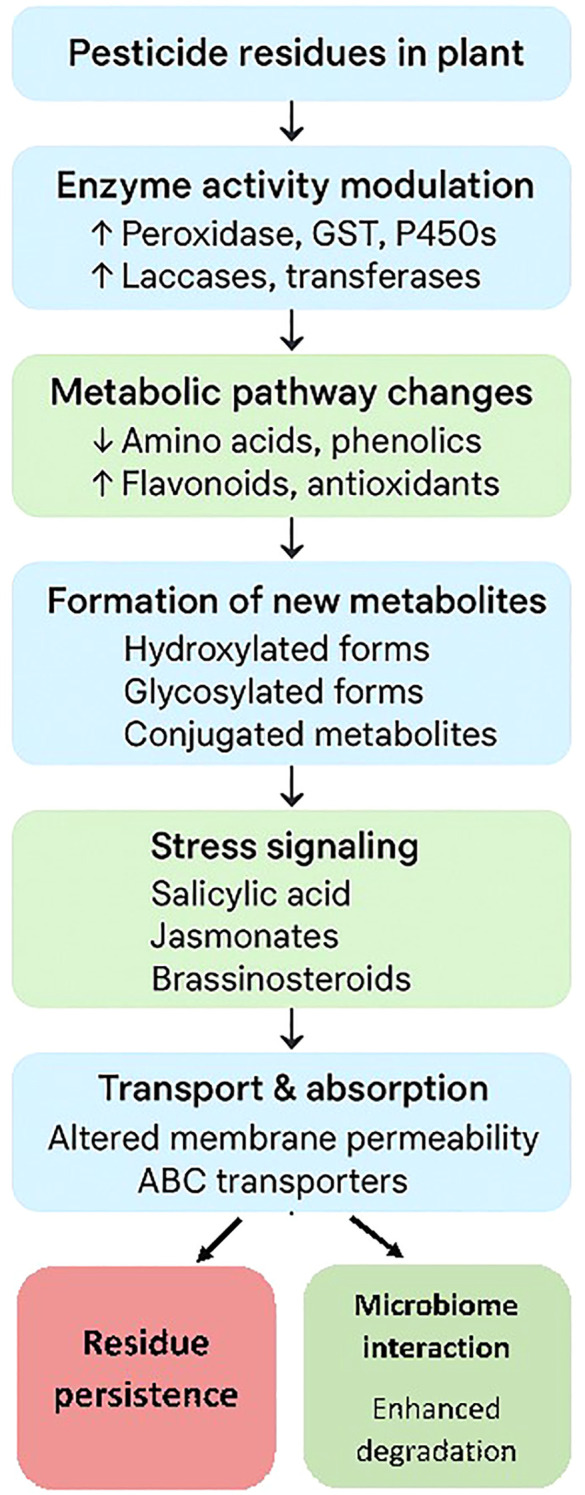
Generalized description of pesticide metabolism in plants.

Plant secondary metabolites play a critical role in modulating pesticide absorption, translocation, and detoxification by enhancing enzymatic breakdown, facilitating conjugation, altering transport processes, and interacting with plant microbiomes. These interactions are central to plant defense and can influence both pesticide efficacy and residue persistence (**Figure 2**).

#### 2.3.1 Phase II metabolism and conjugation

Plant secondary metabolites including flavonoids, alkaloids, and terpenoids are often modified through phase II metabolic processes such as glycosylation, methylation, and hydroxylation. These modifications increase solubility and facilitate storage or transport within plant tissues. Glycosylation, in particular, is a common conjugation reaction, resulting in the formation of glycosides (conjugated metabolites) that are less toxic and more easily compartmentalized in vacuoles or cell walls. Flavonoids, for example, are frequently found as glycosides in plants, and similar conjugation is observed for many alkaloids and terpenoids (Twaij and Hasan, 2022; Elshafie et al., 2023). These plant secondary metabolites (PSMs) play a direct role in the regulation of enzymes involved in pesticide metabolism, including laccases, glycosyltransferases, and methyltransferases. These enzymes are crucial for the detoxification and breakdown of pesticides within plant tissues (Khan et al., 2023; Al-Khayri et al., 2023). Other enzymes that upregulate by PSMs include cytochrome P450s, glutathione *S*-transferases (GSTs), and ABC transporters, which are crucial for breaking down and compartmentalizing pesticides, thereby reducing their toxicity and making them easier to sequester in vacuoles or cell walls, thus limiting their mobility and potential damage (Zhang and Yang, 2021; Kumar et al., 2023; Zhang et al., 2024a).

#### 2.3.2 Stress response and signaling

PSMs are often upregulated in response to pesticide-induced stress, acting as antioxidants and signaling molecules that trigger broader defense responses, including the activation of detoxification pathways (Zhang and Yang, 2021; Elshafie et al., 2023; Khan et al., 2023; Kumar et al., 2023).

#### 2.3.3 Modulation of absorption and translocation

The presence and composition of PSMs can alter the permeability of plant tissues and the activity of transporter proteins, affecting how much pesticide is absorbed and how it is distributed within the plant (Zhang and Yang, 2021; Chang et al., 2024). For example, hydrophobic secondary metabolites may limit pesticide movement by binding or sequestering them in specific tissues (Chang et al., 2024). While the literature extensively discusses plant-bound residues by the formation of conjugated metabolites, direct references to “plant-bound residues” (i.e., metabolites covalently bound to plant macromolecules such as cell wall components) are rare. Most studies have focused on soluble conjugates rather than insoluble, bound forms. However, the structural diversity and reactivity of secondary metabolites suggest that some may form covalent bonds with cell wall polymers or proteins, especially under stress or during defense responses, but this is not well documented in the reviewed sources (Yang et al., 2018; Twaij and Hasan, 2022; Elshafie et al., 2023).

#### 2.3.4 Influence on pesticide efficacy

By modulating the plant’s internal environment, PSMs can affect the persistence and breakdown of pesticides, potentially impacting their effectiveness and residue levels (Zhang et al., 2024b).

#### 2.3.5 Interactions with plant microbiomes

PSMs influence the composition and activity of plant-associated microbiomes, which in turn can affect the degradation of pesticides. Certain microbes are capable of metabolizing both plant-derived secondary metabolites and pesticide residues, sometimes using similar enzymatic pathways. The presence of specific PSMs can select for microbial communities that are more efficient at degrading pesticides, thereby enhancing phytoremediation and biotransformation processes (Pang et al., 2021). The ability of PSMs to modulate pesticide degradation pathways has implications for sustainable agriculture. By engineering or selecting for plants with higher levels of specific secondary metabolites, it may be possible to develop crops that are more effective at detoxifying pesticides, reducing environmental contamination, and improving food safety (Al-Khayri et al., 2023). Additionally, understanding these interactions supports the development of biopesticides and integrated pest management strategies that leverage natural plant defenses (Farhan et al., 2024).

### 2.4 Metabolomics as a complement to residue detection

Routine residue detection methods [e.g., gas chromatography–mass spectrometry (GC–MS), liquid chromatography–tandem mass spectrometry (LC–MS/MS), and immunoassays] provide quantitative data on known pesticide compounds but are limited in scope: they often do not focus on unknown transformation products, conjugated residues, or the dynamic biological impact of exposure. Metabolomics complements these techniques by enabling both untargeted and targeted profiling of small-molecule changes in plants exposed to pesticides. This approach can detect metabolic alterations even when the parent pesticide is below detection thresholds, thereby offering insight into both residue fate and functional stress responses (Mu et al., 2022; Shahid et al., 2023). Recent studies have identified specific metabolite signatures that reliably distinguish pesticide exposure. In rice exposed to chlorpyrifos, Mu et al. (2022) reported significant alterations in 119 metabolites, including increased glutamate-family amino acids, defense-related proline and glutathione, and accumulation of unsaturated fatty acids and phospholipids, while flavonoids were largely downregulated at high doses. Similarly, metabolomics investigations of non-target plant exposure to imazethapyr revealed disruptions in amino acid metabolism and secondary metabolite pathways, providing detailed mechanistic insights into herbicide toxicity (Liu et al., 2024). Broader reviews confirm that metabolomics fingerprints under pesticide stress often involve perturbations in carbohydrate, amino acid, lipid, and phenolic metabolism, sometimes revealing novel degradation products not typically captured by residue analysis (Shahid et al., 2023). The complexity and high dimensionality of metabolomics datasets can easily be tackled with artificial intelligence (AI) and machine learning approaches. Predictive modeling has already been applied to pesticide degradation in soils, where algorithms successfully estimated diazinon residues under varying environmental conditions (Alavi et al., 2022). Adapting similar approaches to plant metabolomics would enable the classification of exposure levels or types based on metabolic fingerprints, the early detection of crop stress prior to visible damage, and the identification of crop genotypes with enhanced detoxification or resilience. The integration of metabolomics with AI-based predictive frameworks therefore holds promise for precision agriculture and food safety.

## 3 Pesticide residues and regulatory framework for food safety

Pesticide exposure to human health can cause respiratory, reproductive, gastrointestinal, and neurological disorders and even cancer (Shekhar et al., 2024). The cytotoxic and mutagenic effects of chemical pesticides can also cause birth defects, reproductive harm, and disruptions in neurological and immune function (Fang et al., 2020). Other negative health effects reported in various studies include acute poisoning and chronic conditions, including different forms of cancer (such as bladder, breast, brain, bladder, and colon cancer) (Rani et al., 2021), Parkinson’s disease (Perrin et al., 2021), Alzheimer’s disease (Frisoni et al., 2022), infertility (Bhardwaj et al., 2020; Foucault et al., 2021), diabetes (Hernández-Mariano et al., 2022), neurotoxicity (Wang et al., 2024a), and leukemia (Rafeeinia et al., 2023). In case of short-term exposure, certain pesticides, especially insecticides, have shown various effects on human health, like dizziness, nausea, skin rashes, and irritated eyes. Breathing problems were reported by farm workers of Ethiopia, Costa Rica, and Brazil (Shah, 2020). Rural Santiago workers exposed to methyl bromide had elevated blood pressure levels and increased mood swings, insomnia, headaches, paresthesia, and memory issues (Zúñiga-Venegas et al., 2022). According to joint reports by the World Health Organization (WHO) and the United Nations Environment Programme (UNEP), it is estimated that approximately three million people suffer from pesticide poisoning annually, with approximately 200,000 deaths, most of which occur in developing countries due to a lack of regulatory oversight and access to protective equipment (W.H.O. and U.N.E.P, 2022). To safeguard public health and food safety, many international and national regulatory bodies have developed maximum residue limits (MRLs) for pesticides. MRLs are the highest level of a pesticide residue that is legally tolerated in or on food or feed when pesticides are applied correctly (F.A.O./W.H.O, 2016). Key organizations involved in setting MRLs include the Codex Alimentarius Commission (CAC) a joint venture of FAO and WHO that provides international food standards, the United States Environmental Protection Agency (EPA), and the European Food Safety Authority (EFSA) (Alimentarius, 2021; European Food Safety Authority (EFSA) et al., 2023; EPA (United States Environmental Protection Agency), 2023). These agencies conduct risk assessments based on toxicological studies and residue trials. The Joint FAO and WHO Meeting on Pesticide Residues (JMPR) plays a critical role in this process. It evaluates scientific data to determine toxicological endpoints, such as the no observed adverse effect level (NOAEL), acute reference dose (ARfD), and acceptable daily intake (ADI) (J.M.P.R, 2023). The JMPR also assesses dietary exposure and provides recommendations to the Codex Committee on Pesticide Residues (CCPR), which finalizes the international standards for MRLs (F.A.O./W.H.O, 2016). Numerous reports have indicated that pesticide residues on food often exceed MRLs (**Table 1**).

**Table 1:** Pesticide residues’ situation compared with MRLs.

Country/region	Samples exceeding MRLs	Most common pesticides detected above MRLs	Sample type/notes	Reference
Turkey	30%	Chlorpyrifos (49 samples exceeded)	Fruits and vegetables	Toptancı et al., 2021
Nigeria (Ikorodu, Lagos)	21.56%	Chlorpyrifos-ethyl (lettuce, spinach), imidacloprid (mangoes)	Vegetables and fruits	Yahaya et al., 2023
Turkey (Aegean region)	11.6%	Chlorpyrifos, azoxystrobin	Fruits and vegetables	Soydan et al., 2021
Sri Lanka	10.1% of fruit samples, 11.6% of vegetable samples, and 41.2% of leafy green samples	Profenofos, chlorpyrifos	Mainly leafy vegetables	Poornima et al., 2024
Egypt (Sharkia Governorate)	40.7% (vegetables), 38.9% (fruits)	Insecticides (general)	Fruits and vegetables	El-Sheikh et al., 2022
China (Fujian)	1.68%	Multiple residues (bananas, peppers highest)	Fruits and vegetables	Zheng et al., 2022
Brazil	67%	Organophosphates, pyrethroids	Review study (systematic)	Andrade et al., 2021
Eastern Mediterranean region	61	Insecticides (general)	Various commodities	Philippe et al., 2021

MRLs, maximum residue limits.

While the analytical studies themselves do not always attribute these exceedances directly to enforcement gaps, complementary regulatory reviews suggest that limited accredited laboratories, weak surveillance systems, informal pesticide markets, and low farmer awareness contribute to inconsistent enforcement of existing MRL standards (Neme et al., 2021; Mahmood et al., 2022; Okoffo et al., 2023). This structural gap implies that the presence of regulations alone does not guarantee compliance; capacity for monitoring and enforcement is critical for practical application. Along with serious health risks, non-compliance with MRLs may result in heavy financial losses. For example, in China, more stringent residue standards led to a 6.6% decline in agricultural export values when MRLs became 10% more restrictive (Zhang, 2025); in Egypt, 52 vegetable export consignments were rejected due to pesticide residues exceeding MRLs, resulting in trade bans (Abd El-Rahman, 2020); in the United Arab Emirates, a survey of imported fruits found MRL exceedances in over 60% of samples from Vietnam and Thailand and over 20% from several other developing countries (Osaili et al., 2022).

Mitigating pesticide residues in agricultural products is crucial for ensuring food safety and environmental protection. Various strategies have been identified to effectively reduce these residues, ranging from household processing techniques to advanced biotechnological methods; for instance, dipping tomatoes in a 2% salt solution can remove up to 76% of certain pesticides (Vemuri et al., 2016). Peeling and blanching can eliminate up to 85% of residues by removing contaminated outer layers (Andreev & Tsygankov, 2022). Advanced treatments, such as cold plasma, ozone application, and ultrasonic cleaning, have shown improved efficacy over traditional methods (Munir et al., 2024). Bioremediation using microorganisms and enzymes is an environmentally friendly option for degrading pesticides in soil and water, especially when enhanced with activated charcoal derived from agricultural waste (Mei et al., 2011). Genetic engineering approaches like CRISPR and RNAi can reduce pesticide dependency by developing pest-resistant crops (Chaudhary et al., 2025). Precision agriculture and integrated pest management (IPM) can further minimize pesticide application by optimizing use and combining multiple pest control strategies (Munir et al., 2024). Vegetated treatment systems, such as constructed wetlands and vegetated ditches, are effective in reducing pesticide runoff, with retention often exceeding 70% (Stehle et al., 2011). Despite the effectiveness of these methods, limitations such as cost, complexity, and incomplete residue removal suggest that combining strategies based on crop type and local conditions is necessary for optimal results.

## 4 Laboratory approach for the analysis of pesticide residues

**Figure 3:**
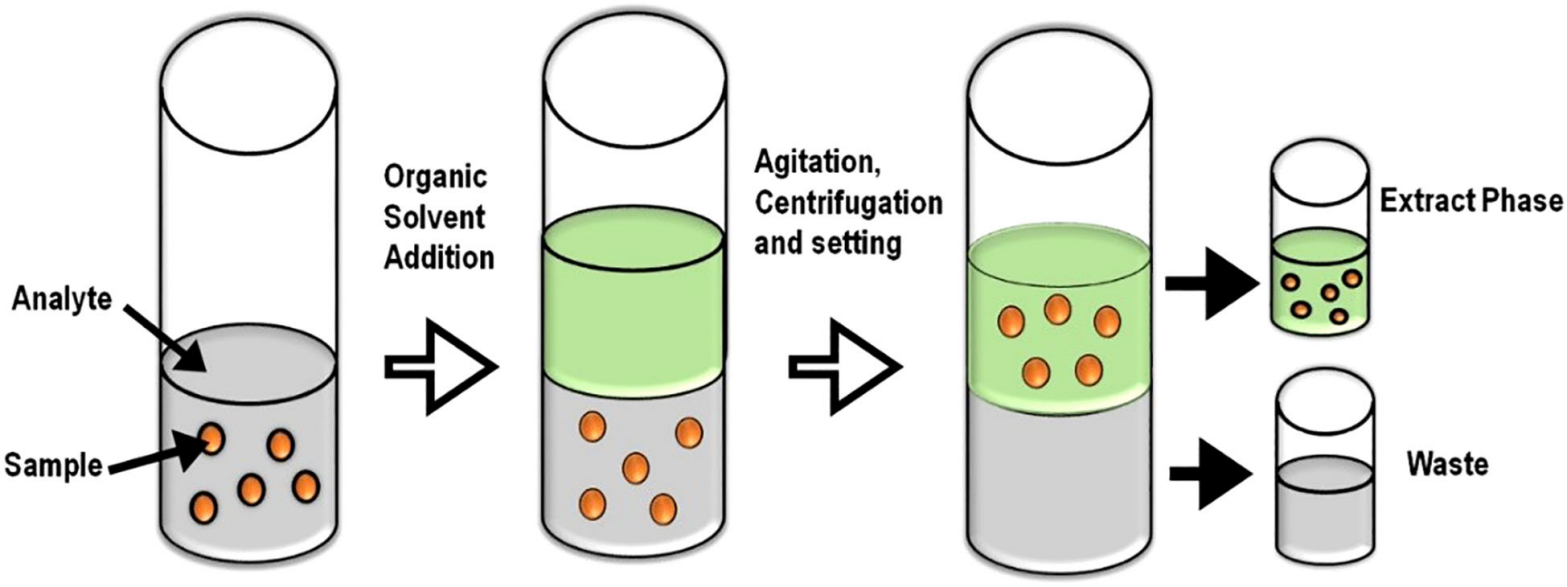
Principle of phase distribution in LLE. LLE, liquid–liquid extraction.

Since regulatory limits of many pesticides are still set at trace amounts, a systematic and careful approach is required. New studies emerge very quickly, and the standardized methods are revised very frequently (**Figure 3**).

### 4.1 Sampling and storage

Sampling is a critical step in pesticide residue analysis that has a direct impact on the accuracy, precision, and reliability of analytical results while extrapolating the results of samples to the whole population. The ultimate aim is to collect a representative portion of crops or fruits that can represent the whole batch or field (Kowalska et al., 2022). Crop type, pesticide application technique, and weather conditions affect pesticide deposition on plant surfaces. Using statistically sound techniques, such as random, stratified, composite, and target sampling, ensures legal and valid results (Enkerlin et al., 2019).

#### 4.1.1 Sampling methods

Different sampling methods are employed based on field size, crop type, and research objectives. Random sampling offers an objective representation of the sample and is employed extensively in quality control analysis and certification audits. Stratified random sampling considers environmental variability, for instance, pest pressure and irrigation heterogeneity, and is ideal for multi-site orchards or experimentation locations. Composite sampling, where multiple samples are merged into one, conserves processing funds and is used most frequently with bulk exports. Its validity decreases if it is not randomized. Systematic sampling, or taking samples at regular intervals across a field, can effectively monitor pesticide distribution trends but is potentially bias-inducing when sample intervals fall in line with application patterns (La Cecilia et al., 2021). Targeted sampling uses selective areas of focus, such as the areas for pesticide sprays or clearly dirty crops, and is convenient to employ for instances of investigation with pesticide misuse but without statistical confidence levels (**Table 2**) (Udomkun et al., 2023).

**Table 2:** Choosing the right sampling for the purpose.

Sampling method	Advantages	Disadvantages
Random	Simple, unbiased representation; widely used in certification and audits	May not capture environmental variability
Stratified	Accounts for field heterogeneity (irrigation, pest pressure)	Requires prior knowledge of variability
Composite	Cost- and time-efficient; suitable for bulk consignments	Reduced statistical validity if not randomized
Systematic	Good for monitoring trends; straightforward implementation	Bias is possible if intervals coincide with spray patterns
Targeted	Useful for investigations and detecting misuse	Not statistically representative

#### 4.1.2 Sample collection procedure

For valid pesticide residue analysis, clean objects such as scissors, knives, or stainless steel blades prevent the contamination of samples. Samples collected in an appropriate quantity should be labelled with information, including crop, collection date, and origin. Crop maturity impacts pesticide uptake, where immature crops have greater residues since they have greater permeability, whereas mature crops have lesser residues because of metabolic hydrolysis (Kariyanna et al., 2024). Weather factors like temperature, humidity, and rain impact residues. Greater temperatures cause rapid degradation, and rain will wash the surface residues off, creating variability. Sampling time is crucial, as pre-harvest and post-harvest pesticide residue concentrations vary according to application timing and the rates of dissipation (Ambrus et al., 2023).

#### 4.1.3 Storage

Sample storage under proper conditions is necessary to conserve pesticide residues and yield accurate analysis (**Table 3**). The storage parameters of significance include temperature, humidity, light, and container material because improper storage may lead to residue degradation, contamination, or loss (Rösch et al., 2023). Inert containers such as glass jars covered with Teflon-lined or high-quality polyethylene bags prevent chemical interference and contamination (Clotea et al., 2021). Maintaining the chain of custody pertaining to sample identification, storage conditions and location, handling person, and the date of time of every step till the samples are discarded after analysis allows traceability and quality assurance. Samples stored in distinction remain safe from cross-contamination as well as exposure to repeated freeze thaw cycles happening while taking out a certain sample for analysis.

**Table 3:** Comparison of different storage conditions and their impact on residue integrity.

Storage condition	Critical time (stability)	Advantages	Disadvantages	Reference
Ambient (room temp.)	<24 h; rapid degradation of dichlorvos, malathion	Convenient, no refrigeration	Significant loss of labile residues	(Bian et al., 2021)
Refrigeration (4°C)	Up to 7 days; stability varies by matrix and pesticide	Minimizes microbial activity, short-term safe	Unstable compounds degrade within days	(Bian et al., 2021)
Freezing (−20°C)	1–3 months; most organophosphates are stable	Suitable for medium-term storage	Some losses in diazinon, matrix-dependent	(Dong et al., 2019)
Deep freezing (−80°C)	6–12 months; stable for most pesticide residues	Best for long-term preservation	High cost, limited access	(Dong et al., 2019)
Pesticide standards mixes	≥12 months (LC–MS/MS and GC–MS/MS), depending on solvent	Reliable calibration reference	Solvent composition affects stability	(Lajin et al., 2023)

LC, liquid chromatography; MS/MS, tandem mass spectrometry; GC, gas chromatography.

### 4.2 Extractions for multi-residue pesticide analysis

#### 4.2.1 Solvent extraction

In a general approach, multiple residues of pesticides are determined after extraction using some efficient solvent extraction methods to purify the residues in matrix-rich foodstuffs. Acetonitrile, hexane, dichloromethane, and ethyl acetate are routinely used organic solvents (Joshi and Adhikari, 2019). Acetonitrile is one of the most frequent solvents employed in liquid–liquid extraction (LLE), which prevents co-extraction of lipids and sugars and is suitable for analysis using LC (Barp et al., 2023; Rudakov et al., 2018). Hexane is commonly employed to extract non-polar pesticides, producing a good recovery of lipophilic pesticide residues. To expand the variety of recovered pesticides, hexane is often mixed with other solvents like ethyl acetate (Negi et al., 2024). Dichloromethane is widely used in both LLE and solid-phase extraction (SPE) since it possesses the ability to dissolve a wide range of pesticide residues. It is especially suitable for the extraction of chlorinated pesticides such as organochlorine (Jones et al., 2023). Ethyl acetate may be used as an alternative to acetonitrile when analyzing multiple residues on GC (Mol et al., 2016). It provides an acceptable recovery of the polar and non-polar pesticides. However, as ethyl acetate is more hydrophobic than acetonitrile, increased co-extraction of background matrix components is more likely in ethyl acetate.

#### 4.2.2 Liquid–liquid extraction

**Figure 4:**
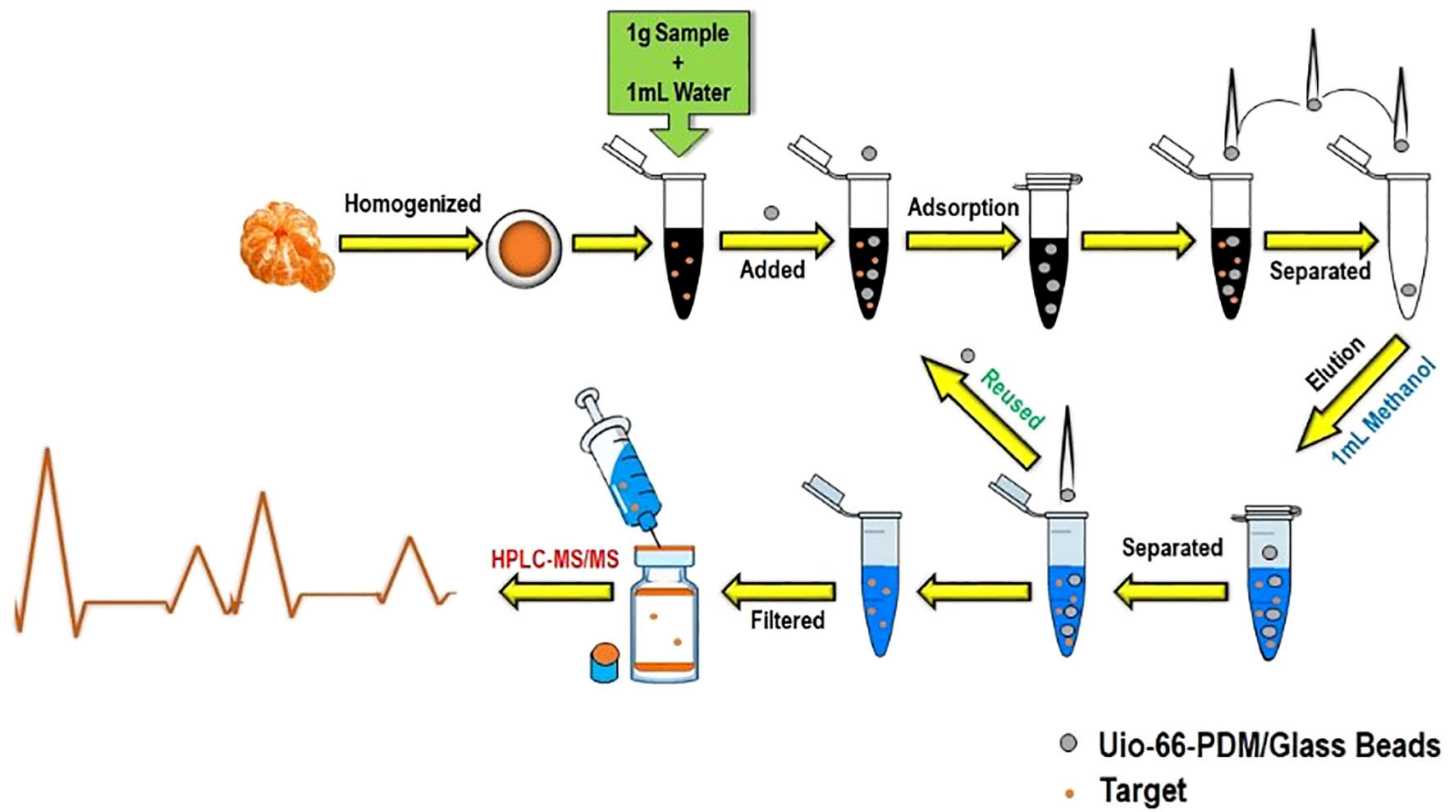
Working flow with UiO-66 (MOF). MOF, metal–organic framework.

LLE is a phase-partition process that separates pesticide residues between two immiscible liquid systems, a polar mostly aqueous phase and a non-polar solvent (such as hexane, ethyl acetate, or dichloromethane) (Chai and Tan, 2016). Despite being a widely used method that is highly efficient in handling non-polar pesticides, LLE is solvent-intensive and environmentally unsustainable in comparison to newer alternatives (**Figure 4**) (Campanale et al., 2021); thus, it is increasingly being replaced by newer alternatives such as Quick, Easy, Cheap, Effective, Rugged, and Safe (QuEChERS) and SPE, offering better selectivity and low solvent consumption (Mandal et al., 2023).

#### 4.2.3 Solid-phase extraction

SPE is a process that employs solid sorbents [C18, primary secondary amine (PSA), and graphitized carbon black (GCB)] to retain pesticides, excluding matrix interferences (Biziuk and Stocka, 2015a). The process is beneficial in the sample cleanup of samples with low matrix effect (Besil et al., 2017). SPE is a more accurate, solvent-saving process and, hence, a better process for targeted analysis of very dilute samples (Ramadevi and Ramachandraiah, 2023). It is particularly effective for selectively retaining pesticides, which can then be eluted with appropriate solvents. SPE is suitable for automation and integration with LC or GC systems. When combined with other techniques like dispersive liquid–liquid microextraction (DLLME), SPE enhances extraction efficiency and preconcentration factors (Shamsipur et al., 2016). The integration of green solvents and magnetic sorbents in SPE methods offers environmentally friendly and cost-effective solutions for pesticide extraction from food products (Zhao et al., 2019; Wang et al., 2021b).

#### 4.2.4 QuEChERS extraction

The QuEChERS (Quick, Easy, Cheap, Effective, Rugged, and Safe) method is a widely used extraction technique initially developed for pesticide residue analysis in fruits and vegetables. QuEChERS has been extensively applied beyond its original scope of pesticide analysis. It is now used for detecting pharmaceuticals, polycyclic aromatic hydrocarbons (PAHs), persistent organic pollutants (POPs), and other contaminants in food, biological, and environmental samples (Kim et al., 2019; Musarurwa et al., 2019; Perestrelo et al., 2019). The method’s adaptability is further demonstrated by its use in diverse fields such as forensic analysis, environmental monitoring, and doping control (Perestrelo et al., 2019; Derwand et al., 2023). Modifications to the original protocol have been made to enhance extraction efficiency for specific analytes and matrices. These include the use of different solvents, salt formulations, and sorbents, as well as the incorporation of steps like alkaline hydrolysis and freeze-out for specific sample types (Kim et al., 2019; Musarurwa et al., 2019; Acosta-Dacal et al., 2021). Compared to LLE, QuEChERS is cost-effective and time-saving and provides higher recovery rates and better analytical performance, often eliminating the need for concentration steps (Kim et al., 2019; Perestrelo et al., 2019; Shin et al., 2022).

#### 4.2.5 Environmentally sustainable extraction techniques

To minimize solvent consumption and maintain the sustainability of the environment, new approaches to extraction, like 1) supercritical fluid extraction (SFE) and 2) ultrasound-assisted extraction (UAE), are being explored (Gaikwad et al., 2024). UAE utilizes sonication to allow the penetration of the solvent in solid matrices (Nguyen et al., 2020), reducing the time of extraction without sacrificing efficiency. As a green approach to extraction, SFE utilizes a solvent of supercritical CO_2_, offering a promising alternative to toxic organic solvents (Arumugham et al., 2021) (**Table 4**).

**Table 4:** Comparison of extraction techniques and solvents used.

Method	Solvent usage	Selectivity	Automation	Matrix effects	Environmental impact	Usages	References
LLE	High (hexane, dichloromethane, ethyl acetate)	Low to medium	Low	High	High (toxic solvents)	Non-polar pesticides	(Taylor et al., 2002)
SPE	Low (methanol, acetonitrile, water-based solvents)	High	High	Very low	Low	Selective pesticide analysis, cleanup	(Biziuk and Stocka, 2015a)
QuEChERS	Medium (acetonitrile)	High	Moderate	Low	Medium	General pesticide screening	(Yuan et al., 2024)
UAE	Low (water, ethanol, acetone)	High	Moderate	Low	Low	Green alternative, rapid screening	(Nguyen et al., 2020)
SFE	Very Low (supercritical CO_2_)	High	High	Low	Very low	Green chemistry, sensitive analysis	(Saito-Shida et al., 2014)

LLE, liquid–liquid extraction; SPE, solid-phase extraction; QuEChERS, Quick, Easy, Cheap, Effective, Rugged, and Safe; UAE, ultrasound-assisted extraction; SFE, supercritical fluid extraction.

#### 4.2.6 Emerging extraction techniques and modifications

**Figure 5:**
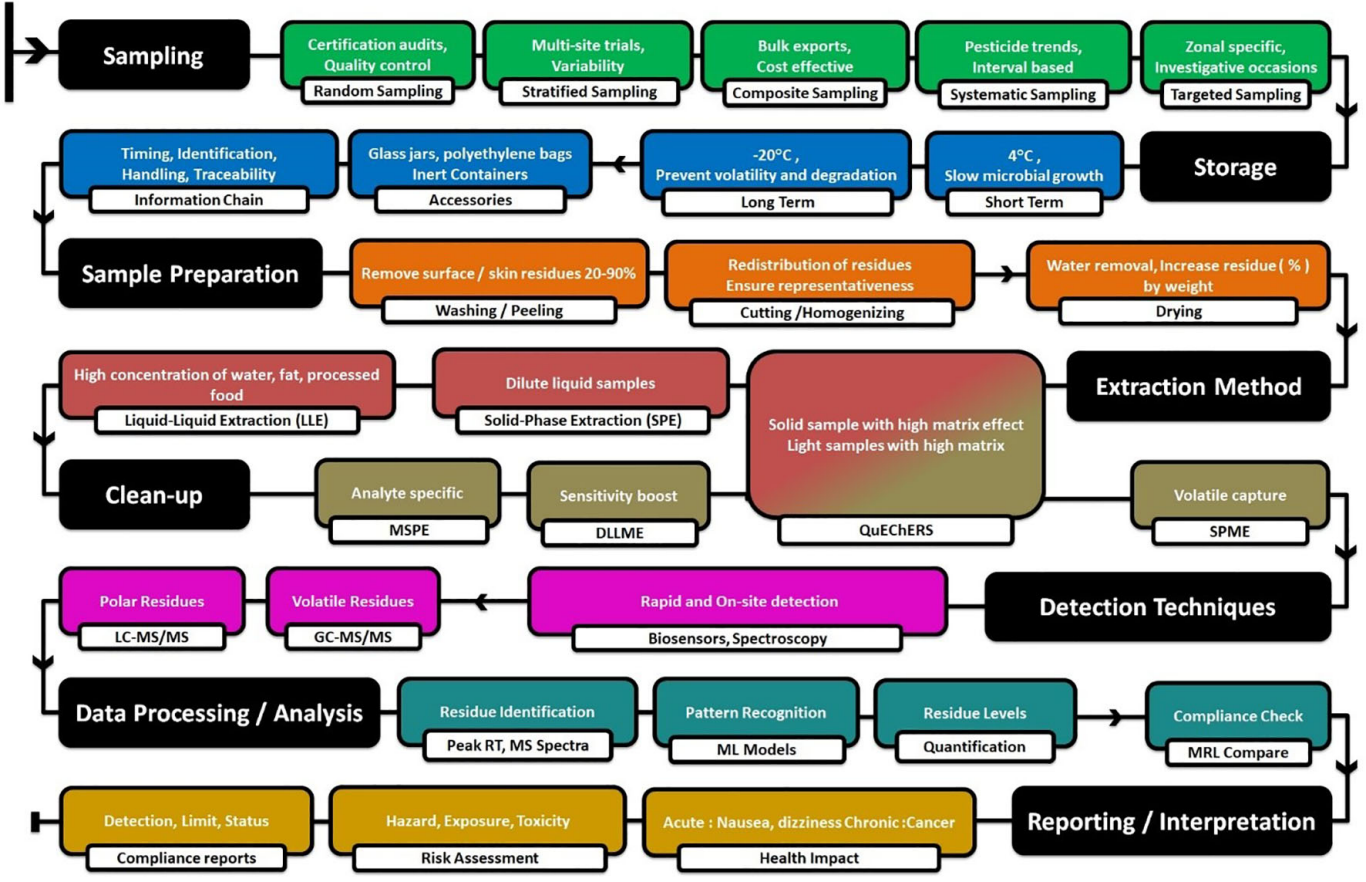
Schematic description of pesticide residue analysis.

Recent advancements and the coupling of innovative strategies with classical extraction and separation techniques have led to significant improvements in analytical efficiency, precision, and environmental sustainability, allowing laboratories to navigate increasingly complex sample matrices and regulatory requirements with greater reliability. The QuEChERS method has seen widespread adoption in food and soil analysis, as it is flexible to work with both gas and liquid chromatography systems. QuEChERS coupled with magnetic solid-phase extraction (MSPE) enables enhanced selectivity and simplified separation (Du et al., 2019). Further advancements incorporate solid-phase microextraction (SPME) for ultra-low volatile and semi-volatile compounds (García-Reyes, 2022), salting-out assisted liquid–liquid extraction (SALLE), and molecularly imprinted polymers (MIPs) (Hashemi, 2018). The integration of QuEChERS with SPE or DLLME has demonstrated significant reductions in matrix interference while achieving consistently high recovery yields across multiple classes of compounds (Biziuk and Stocka, 2015b; Pastor-Belda et al., 2016). Moreover, citrate-buffered AOAC 2007.01 and CEN EN 15662 protocols validated for LC–MS/MS and GC–MS/MS applications offer streamlined workflows for high-throughput multi-residue analysis in regulatory and research environments (Anastassiades et al., 2023; Fernández-Alba, 2023). These methods are also increasingly used in parallel with performance verification experiments, validating their robustness against other methodologies. SFE, when optimized with co-solvents such as ethanol and methanol, greatly enhances the recovery of polar analytes (Liu, 2021). Particularly well-suited for the extraction of thermolabile and non-polar compounds, SFE maintains analyte integrity while operating under mild thermal conditions. Its synergy with solid-phase techniques or post-processing using DLLME is being explored to expand its capability into broader pesticide classes. Similarly, microwave-assisted extraction (MAE) and UAE show promise in processing samples with complex matrices such as plant tissues or composite food products, offering accelerated extraction with reduced solvent volumes and energy input (Nguyen, 2020). The combination of MSPE with innovative sorbents such as metal–organic frameworks (MOFs) (**Figure 5**), graphene-based composites, or covalent organic frameworks (COFs) provides high-efficiency options for automation, minimal sample preparation, and low-solvent usage (Díaz-Álvarez, 2019; Yang et al., 2023), making it attractive for automated laboratories and high-throughput screening environments. In addition, the evolution of magnetic nanoparticles has significantly improved reproducibility and operational simplicity, especially when used in combination with QuEChERS workflows (Du et al., 2019).

UAE’s integration with ionic liquids (ILs) or deep eutectic solvents (DESs) is proving effective in improving recovery and selectivity while maintaining environmental compatibility (Flores, 2024). UAE is also increasingly being used in tandem with pre-concentration steps like SPE or DLLME to ensure sufficient enrichment of trace-level pesticide residues. DLLME continues to be favored for its rapid processing time, high enrichment factors, and low solvent consumption. It is especially effective for polar and semi-polar analytes in aqueous matrices, where its unique dispersive mechanism allows intimate interaction between extractant and target compounds. The introduction of DES or task-specific ILs into DLLME protocols is expanding its applicability to more complex sample types and improving its compatibility with greener workflows (Hashemi et al., 2018). In pharmaceutical applications, coupled systems such as SPE–SFE or SPME–GC–MS are used for drug profiling, bioavailability assessments, and impurity detection. These hybrid techniques allow simultaneous extraction and clean-up steps, which improve analytical turnaround times while reducing matrix interferences. DLLME has emerged in environmental monitoring of pharmaceutical residues, especially in wastewater matrices, where traditional SPE systems may face challenges in enrichment and recovery (Pastor-Belda et al., 2016). The application of QuEChERS with MIPs also strengthens food quality assessment protocols by improving analyte specificity and reducing the risk of false positives when screening for mycotoxins, heavy metals, or pesticide residues (Hashemi, 2018; Anastassiades et al., 2023). In environmental applications, methods such as SFE, UAE, and SPME have proven to be adaptable for the extraction of contaminants, including POPs, heavy metals, and pharmaceutical residues from soil, water, and sediment matrices (Nguyen, 2020; García-Reyes, 2022). These techniques are particularly relevant in regions where regulatory frameworks require the simultaneous screening of multiple classes of contaminants with varied physicochemical properties. Optimization strategies in modern extraction workflows focus on critical variables such as solvent composition, sample pH, ionic strength, and temperature control. For example, optimizing the disperser-to-extractant ratio in DLLME can significantly improve analyte recovery, especially in complex matrices (Pastor-Belda et al., 2016). Likewise, sorbent choice in SPE whether using classical C18, PSA, or more advanced MOF or MIP materials determines the specificity and efficiency of isolation (Díaz-Álvarez, 2019). Automation and AI are increasingly integrated into extraction workflows to optimize solvent selection, extraction conditions, and procedural timing. AI models help streamline method development and allow adaptive adjustments based on real-time feedback from analytical instruments (Liu, 2021). Robotics is being adopted for sample preparation tasks, significantly enhancing reproducibility and analytical throughput, especially in high-volume laboratories (Flores, 2024). The trajectory of modern separation science is toward blending sustainability with high analytical performance. Functionalized nanomaterials are being synthesized for targeted selectivity, while 3D-printed microfluidic devices and lab-on-chip platforms are being developed for *in situ*, field-level extractions (García-Reyes, 2022). These advancements aim to make residue testing more accessible and scalable. As global focus intensifies on reducing laboratory waste and environmental impact, regulatory bodies are aligning with green chemistry principles. Solvent minimization, recyclability, and the implementation of biodegradable alternatives such as DES and ILs are becoming standard benchmarks for method approval (Zhang et al., 2018a).

##### 4.2.6.1 Stability during extraction

Removal of multiclass pesticides in fruits, such as citrus, has some major stability concerns that should be handled properly. The high pigment composition, essential oils, and organic acids in citrus fruit matrices provide a complex composition that can greatly impact pesticide analysis stability (Mol et al., 2019). The stability of these matrix compounds is likely to be sub-optimal due to co-extraction with the target pesticides. The QuEChERS-based method has been the most feasible method for stability during multiclass pesticide extraction as a process. The original technique of Pszczolińska and Kociołek (2022) has been used in applications similar to matrix effect reduction using PSA, GCB, and C18 sorbent. The standard solvent extraction method using organic solvents exists only in the embryonic phase because it faces severe stability problems. For example, Lee et al. (2024) noted that solvents such as acetonitrile, ethanol (different grades), ethyl acetate, and methanol are general extraction solvents for pesticides; however, their stabilizing properties greatly deteriorate in the presence of mixtures of residues (Koch et al., 2020). It can clearly be observed that when more than five analytes are to be analyzed simultaneously, the possible occurrence of concurrent extraction of matrix-interfering compounds becomes more likely. Improved extraction techniques have shown recent advances that emerge with a miniaturized version (Tang et al., 2019). MSPE and Dispersive Solid-Phase Extraction (d-SPE) are highly selective; however, d-SPE does not optimize pesticide integrity when solvent use is reduced (Zhang et al., 2018a, b). Natural deep eutectic solvents (NADESs) and DESs show biocompatibility, which allows enhanced stability in the enhancement of extraction. Tang et al. (2019) also improved the ability of ILs. Benzimidazole conjugates need to be handled properly to retain their fluorescence, a prerequisite for detection (Bajwa et al., 2017). This kind of degradation makes neonicotinoid a significant analytical challenge, and special liquid–liquid extraction has to be used in order to retain the stability of metabolites, hence emphasizing liquid–liquid as the only suitable procedure at trace concentrations (Casado et al., 2018). Wang et al. (2021a) demonstrated that the development of stability in the extraction step relies on a multi-principled approach, like the use of specific sorbent pairs for matrix dispersal, the miniaturization of approaches when possible, and the adaptation of extraction protocols to cater to the type of pesticide. Analytical challenges in multi-residue citrus fruit extraction arise from individual matrix characteristics. When the whole fruit and vegetables are analyzed, the high sugar percentage, as well as the presence of 108 carotenoids, can create a serious competition interference in the determination of pesticides (Mol et al., 2019). Matrix interference confirms that ionization in LC–MS/MS or GC–MS analyses can be greatly affected by the matrix components discussed above, leading to falsely identified results (Wang et al., 2021a).

#### 4.2.7 Exceptions and challenges

The high-water content of citrus fruits presents special extraction challenges. According to Goel et al. (2025), aqueous-based extraction processes will likely lose fat-soluble or non-polar pesticides since the majority of pesticides are in trace amounts or are bound to water-soluble compounds. Pesticide residues will likely separate differently in citrus pulp and peel, with peel having greater concentrations due to direct exposure. Zhang et al. (2018b) also explained that conventional extraction methods can fail to recover residues in both fractions; therefore, various extraction methods have to be applied. Storage conditions also significantly impact the stability of the pesticide, as Casado et al. (2018) proved that heat, light, or oxygen can lead to degradation and subsequently underestimate the concentrations of residues. Zhang et al. (2018a) said that analysis of very dilute samples doubles the time and complexity of extraction and analysis procedures. The varied range of chemical properties of pesticides, including polarity, volatility, and solubility, makes the process complicated. Tang et al. (2019) clarified that there is no single technique to successfully extract all types of pesticides and that each category of chemicals needs a unique procedure. Operations after extraction are a further problem. Bajwa et al. (2017) outlined how the currently leading methodologies involve tedious clean-up procedures to remove interfering agents like pigments, waxes, and organic acids, with multiple steps in purification needed. The regulatory framework contributes to the problem, as Casado et al. (2018) clarified that various jurisdictions have varying maximum residue levels for pesticides, which influence the level of sensitivity needed. Recent technological advancements through 2025 have introduced improved ways to address these limitations.

### 4.3 Analytical techniques for the detection of pesticide residues

The ultimate goal of the extraction is to remove unwanted matrix and maximize the target analyte concentration to enhance the detection. After the careful extraction, various analytical methods used for the detection of trace analytes can also be employed for the detection of pesticide residues (**Figure 3** and **Table 5**). The methods can be separative and non-separative. Among the separative methods, chromatographic techniques, especially gas chromatography and liquid chromatography, are widely used.

**Table 5:** Quick overview of non-separative analytical techniques.

Technique	Typical detection limit	Reproducibility	Field feasibility	Reference
Raman spectroscopy	μg/cm^2^–ng/cm^2^ on surfaces	Moderate; dependent on signal strength and preprocessing	High; handheld Raman available	(Ndung’u et al., 2024b)
SERS	nM–pM; ng/cm^2^ on surfaces	Low; substrate and solvent variability	High; portable, flexible substrates	(Wei et al., 2023; Averkiev et al., 2024a; Fǎlǎmaş et al., 2024b)
2D-COS Raman + chemometrics (PCA, SVM)	Classification accuracy up to 100%	High; improved robustness with preprocessing	High; compatible with portable Raman	(Ndung’u et al., 2024b)
Fluorescence + CNN	High classification accuracy; LOD analyte-dependent	Moderate; subject to quenching/interference	Moderate; some portable systems	(Wang et al., 2023b)
Biosensors	nM–pM; example: paraquat 2.76 nM	High within batch; stability issues long-term	High; low-cost, point-of-use	(Wu et al., 2022; Frontiers, 2023)

SERS, surface-enhanced Raman spectroscopy; 2D-COS, two-dimensional correlation spectroscopy; PCA, principal component analysis; SVM, support vector machine; CNN, convolutional neural network; LOD, limit of detection.

#### 4.3.1 Gas chromatography

GC is particularly effective for volatile and semi-volatile, thermally stable compounds, allowing for the separation of pesticide residues from complex matrices (Meher and Zarouri, 2025). The technique relies on the vaporization of samples and their subsequent passage through a column, where they are separated based on their affinity to the stationary phase. The separated compounds are then detected using a suitable detector (Amórtegui and Dallos, 2018). The choice of column type and detector is dependent upon several factors, such as the target analyte, nature of samples, resolution and sensitivity required, and financial budget. While the column affects the separation efficiency and detection sensitivity, the detector can significantly influence the sensitivity and specificity of the analysis.

##### 4.3.1.1 Commonly used GC column types

Capillary columns with narrow diameter and long length, typically coated with stationary phases like polydimethylsiloxane (non-polar) or polyethylene glycol (polar), offer high resolution (Kanateva et al., 2021). Non-polar columns such as DB-1, HP-1, and Rtx-1 are suitable for analyzing less polar compounds, such as organochlorines and pyrethroids (Picó et al., 2020). Columns with polar stationary phases, like DB-WAX and HP-INNOWax (polyethylene glycol), are used for polar pesticides such as organophosphates and carbamates (Torres et al., 2021). In general, partially polar columns such as DB-5, HP-5, and Rtx-5 are used in response to various pesticides. Both the column and detector are paired accordingly. For instance, electron capture detection (ECD) is often paired with non-polar columns for organochlorine pesticides, while nitrogen–phosphorus detection (NPD) may be used with polar columns for organophosphates (Radović et al., 2015; Anagnostopoulos et al., 2015). Column operations, such as temperature ramping, gas pressure, and flow rate, are also crucial for effective separation and method run time. Hakme et al. (2018) developed a multi-residue method using GC coupled with triple quadrupole mass spectrometry (GC–QqQ–MS/MS) capable of analyzing 203 pesticides in a single run of 12.4 minutes, with 2 μg/kg Limit of Quantification (LOQ). Adjusting the carrier gas pressure and flow rate is also essential for achieving optimal separation and peak resolution.

##### 4.3.1.2 Common detectors for GC

There are many selective detectors such as ECD, nitrogen–phosphorous detector (NPD), flame photometric detector (FPD), and flame ionization detector (FID). ECD is highly sensitive to electronegative compounds, such as organochlorine pesticides, so it is particularly effective for detecting chlorpyrifos, cypermethrin, and other organochlorines, showing good linearity with correlation coefficients above 0.99 (Sofi et al., 2023). Using ECD methods, Yu et al. (2021) demonstrated the recoveries for organochlorines in vegetables ranging from 78.8% to 92.2%, with relative standard deviations (RSDs) generally below 10%. Similarly, in fruit analysis, average recoveries were above 90% with RSDs less than 6% (Tankiewicz, 2019). In a method for fipronil and its metabolites, the limits of detection (LODs) have been reported as 0.0005 mg/L for the metabolites and 0.0003 mg/L for fipronil, while the LOQs were established at 0.002 mg/kg for all compounds across maize grain, maize stem, and soil, which is below the established tolerance levels in the USA and European Union (EFSA, 2023). FID is often considered a universal detector due to its broader range of detection. However, its sensitivity is generally lower compared to that of ECD for electronegative compounds (Lopes et al., 2022). Its response is more dependent on the structure of compounds; generally, FID is best with hydrocarbons and other non-polar compounds (Spanjers et al., 2017). The ionization energy and molecular orbital energy are key factors influencing FID’s response (Lopes et al., 2022). FID methods for pesticide analysis have been showing average recoveries ranging from 78.67% to 85.1% for organochlorine pesticides, with method detection limits typically in the range of 0.048 to 0.089 µg/mL (Gaber, 2014). The dielectric barrier discharge ionization detector (BID) has been shown to have a greater response to pesticides compared to FID, with relative responses of approximately 3.0, a promising alternative for comprehensive pesticide analysis (Lopes et al., 2022). The combination of FPD and ECD in a single setup allows the simultaneous detection of different pesticide classes in residue analysis (Saha et al., 2020). Finally, the integration of mass spectrometry with GC (GC–MS) offers high sensitivity, with the detection of pesticide residues at levels as low as 0.05 to 1 mg/kg (Ali and Hassan, 2023), and as low as 2 μg/kg when used with triple quadrupole MS (GC–QqQ–MS/MS) (Hakme et al., 2018). Further, the identification of compounds based upon their mass spectra enhances the specificity of GC–MS (Wilson-Frank and Ewbank, 2022). The technique is adaptable to different sample preparation methods and is applicable to diverse food matrices (Dar et al., 2023). The ability of GC–MS to perform both targeted and non-targeted analyses allows the detection of known pesticides and the identification of unexpected compounds or metabolites, enabling the comprehensive monitoring of pesticide residues (Chou et al., 2023; Su et al., 2024). The technique can work with fast temperature ramping for high-throughput screening while remaining robust and reliable and showing good linearity (Dar et al., 2023; Romagnoli et al., 2023; Su et al., 2024). Advanced GC–MS technologies, such as MS/MS, has been shown to detect over 200 compounds in a run time of 30 minutes (Kim et al., 2024). Time of flight (TOF) with GC–MS enables the detection of over 1,000 pesticides without the need for expensive analytical standards (Chou et al., 2023). Despite the growing preference for LC–MS/MS for certain pesticide classes, GC has remained a robust and cost-effective option for many applications (Brandi et al., 2024).

#### 4.3.2 Liquid chromatography–mass spectrometry

LC–MS is another powerful analytical technique widely used for detecting pesticide residues in various matrices. When combined with QuEChERS extraction, it is capable of simultaneous detection of multiple pesticides. Average studies of LC–MS/MS method development are upcoming with the detection of 35–50 pesticides, with recoveries ranging from 80% to 90% (Majumder et al., 2024). An LC–High-Resolution Mass Spectrometry (HRMS) method optimized for analyzing 18 pesticides in river water and seawater achieved quantification limits as low as 1.7 ng/L (Nannou et al., 2024). SPME combined with LC–MS/MS was used for detecting organophosphorus pesticides in tea, offering a good linear range, low detection limits of 0.01 µg/kg, and high recovery rates of over 90% (Meng, 2024). Another study validated a method for detecting over 1,100 pesticides and toxins in food, highlighting its capability to meet stringent regulatory requirements (Bessaire et al., 2024). Abo-Gaida et al. (2022) utilized LC–MS/MS to monitor acidic pesticide residues in various water sources in Egypt, with LOD as low as 0.5 ng/L, demonstrating the method’s reliability for environmental monitoring.

##### 4.3.2.1 Supercritical fluid chromatography–mass spectrometry

Supercritical fluid chromatography–MS (SFC–MS) is a chromatographic technique that uses supercritical CO_2_, sometimes modified with organic solvents like methanol, as the mobile phase. It combines gas-like diffusivity and liquid-like solvating power, enabling fast and efficient separation of non-polar to moderately polar compounds. SFC–MS offers a more environmentally friendly alternative by reducing solvent use and analysis time, thus reducing cost in routine analysis of pesticides in grains and other agricultural products (Xie and Zhang, 2023). Further, the development of green analytical chemistry and the integration of advanced technologies like hyperspectral imaging and machine learning are also paving the way for more sustainable and efficient pesticide detection methods in the future (Kaur et al., 2021; Chaichana et al., 2023).

##### 4.3.2.2 Non-separative testing methods

Certain detectors can detect the compounds without a prior separation; however, their results are a subject of discussion. Non-separative techniques (**Figure 3** and **Table 6**) for pesticide residue analysis are gaining traction due to their ability to provide rapid, cost-effective, and non-destructive assessments, as well as advantages over traditional separative techniques, such as reduced sample preparation time, minimal waste generation, and the developing ability to analyze complex mixtures (Gai et al., 2023). These include chemical spot tests using Ellman’s reagent (DTNB), which are destructive in nature, but other tests can be non-destructive (Cao et al., 2025). Non-destructive testing (NDT) techniques allow the simultaneous measurement of chemical and physical characteristics without damaging the sample. However, NDT methods face challenges in implementation, such as identifying mixed pesticides and performing volumetric quantification beyond surface accumulation (Sindhu and Manickavasagan, 2023). Their detection capabilities have been shown to increase profoundly when subjected to analysis after matrix removal and clean up by QuEChERS or SPE methods (Norli et al., 2015; Rahman et al., 2017).

**Table 6:** Analytical methods published for analyzing pesticide residues in previous 10 years.

Sr. no.	Analytes	Matrix	Extraction method	Detection	LOD	LOQ	RSD (%)	References
1	8 multiclass pesticide residues	Orange, lemon (juices)	Magnetic Stir Bar–Liquid Phase Microextraction (MSB–LPME)	GC–MS	0.000018–0.000096 μg/mL	0.00006–0.00032 μg/mL	<6.1	(Wu et al., 2015)
2	19 multiclass pesticide residues	Orange (juice)	SPE + DLLME	GC–MS	5.0 × 10^−7^–1.0 × 10^−6^ µg/g	–	0.8–10	(Shamsipur et al., 2016)
3	Triazoles	Tea	d-SPE–DLLME	LC–MS/MS	4 × 10^−6^–3.16 × 10^−5^ µg/g	–	<14	(Zhang and Xu, 2014)
4	360 multiclass pesticide residues	Orange (pulp)	QuEChERS	GC–MS/MS	–	0.001–0.05 μg/g	≤20	(Lee et al., 2018)
5	439 multiclass pesticide residues	Fruits + vegetables	QuEChERS	GC–MS	–	0.001–0.0015 μg/g	<20	(Li and Jenninngs, 2017)
6	Fluazinam	Citrus	QuEChERS	GC–ECD	0.003 μg/mL	0.01 μg/g	<5.7	(Zhao et al., 2021)
7	7 multiclass pesticide residues	Pomegranate, orange (juice)	d-SPE + DLLME	GC–MS, FPD	0.0008–0.00116 μg/mL	0.0000028–0.000004 μg/mL	<8	(Farajzadeh et al., 2020)
8	9 multiclass pesticide residues	Pomegranate, orange (juice)	d-SPE + DLLME	GC–FPD	0.00032–0.00076 μg/g	0.0011–0.0026 μg/g	<8.4	(Mohebbi et al., 2020)
9	Chlorpyrifos, hexaconazole	Orange (juice)	d-SPE	GC–ECD	0.00067–0.00089 μg/mL	0.00222–0.00294 μg/mL	<5.6	(Asadi‐Sabzi et al., 2020)
10	7 multiclass pesticide residues	Orange (juice)	SPE + DLLME	GC–FPD	0.00030–0.00061 μg/g	0.0010–0.0020 μg/g	3.0–6.0	(BakhshizadehAghdam et al., 2021)
11	6 multiclass pesticide residues	Pomegranate, orange (juice)	DLLME	GC–FID	0.00030–0.00061 μg/g	0.0010–0.0020 μg/g	2.2–5.8	(Farajzadeh et al., 2020)
12	Fenitrothion, malathion, ethion, chlorpyrifos, diazinon	Orange (juice)	Dispersive Solid-Phase Microextraction (DSPM)	GC–FID	0.00003–0.00011 μg/mL	0.00011–0.00038 μg/mL	<4.59	(Ghorbani et al., 2021)
13	115 multiclass pesticide residues	Orange (whole fruit)	QuEChERS	High-Performance Liquid Chromatography (HPLC)–MS/MS	0.000001–0.000007 μg/g	0.000003–0.000019 μg/g	≤20	(Golge & Kabak, 2015)
14	200 multiclass pesticide residues	Orange (whole fruit)	Methanol extraction	HPLC–MS/MS	–	0.000010–0.000100 μg/g	≤25	(Hanot et al., 2015)
									15	Etoxazole	Orange (whole fruit)	QuEChERS	HPLC–MS/MS	–	0.005 μg/g	0.8–5.4	(Yao et al., 2015)
16	Multipesticides	Orange (whole fruit)	MSPE	HPLC–UV	0.0097–0.010 μg/g	0.039–0.32 μg/g	<10	(Gao et al., 2015)
17	Neonicotinoid pesticide residues	Orange (juice)	LLME	HPLC–UV	0.00025–0.00030 μg/mL	0.00150–0.00180 μg/mL	2.7–5.4	(Vichapong et al., 2016)
18	Spirodiclofen–pyridaben	Citrus (whole fruits)	Acetonitrile (ACN) extraction	HPLC–MS/MS	0.001 μg/g	0.005 μg/g	NR	(Sun et al., 2016)
19	256 multiclass pesticide residues	Lemon (essential oil)	Dilution method + evaporation under nitrogen	HPLC–MS/MS	–	≤0.010 μg/mL	<5	(Fillatre et al., 2017)
20	74 multiclass pesticide residues	Orange (pulp)	QuEChERS	HPLC–MS/MS	0.0003–0.067 μg/g	0.001–0.222 μg/g	<19	(Rizzetti et al., 2016)
21	Chlorpyrifos, carbendazim	Tangerine, grapefruit (whole fruits)	QuEChERS	HPLC–FD	0.6–0.7 μg/g	0.19–0.22 μg/g	0.4–0.5	(Hazer et al., 2017)
22	Carbendazim, thiabendazole	Lemon (whole fruit)	SPE	HPLC–UV	0.00045–0.00054 μg/mL	0.0015 0–0.00180 μg/mL	1.3–3.9	(Wang et al., 2016)
23	5 multiclass pesticide residues	Apple, grapes, peach, kiwi, orange (whole fruit)	Modified QuEChERS	HPLC–MS/MS	0.000003–0.00018 μg/g	0.00001–0.00059 μg/g	0.57–12	(Yan et al., 2018)
24	5 multiclass pesticide residues	Orange (whole fruit)	QuEChERS	UHPLC–MS/MS	–	0.0001–0.0015 μg/g	<17	(Li et al., 2017)
25	93 multiclass pesticide residues	Orange (whole fruit)	QuEChERS	GC, HPLC–MS	–	<0.005 μg/g	NR	(Suárez-Jacobo et al., 2017)
26	Thiabendazole	Citrus, lemon (whole fruits)	MSPE	HPLC–UV	0.004–0.009 μg/g	–	<4	(Díaz-Álvarez and Martín-Esteban, 2018)
27	Thiacloprid	Citrus (whole fruits)	SALLE	HPLC–Diode Array Detector (DAD)	0.03 μg/mL	0.05 μg/mL	3.0–5.0	(Akram et al., 2018)
28	Novaluron, pyriproxyfen, thiacloprid, tolfenpyrad	Citrus (whole fruits)	SPE	HPLC–MS/MS	0.00001–0.0008 μg/mL	0.005 μg/g	<6.7	(Dong et al., 2018)
									29	8 multiclass pesticide residues	Orange (whole fruit)	QuEChERS + d-SPE + DLLME	HPLC–MS/MS	0.00002–0.00032 μg/g	0.00007–0.00106 μg/g	<18	(Lawal et al., 2018)
30	165 multiclass pesticide residues	Citrus (peel, albedo, pulp)	Solvent extraction	HPLC–MS/MS	–	0.71–5.97 μg/g	<20	(Calvaruso et al., 2020)
									31	Carbendazim	Orange (juice)	Dispersive Pipette Extraction (DPX)	HPLC–DAD	8.7–15 μg/mL	102–110 μg/mL	<16	(de Aguiar et al., 2020)
32	Carbendazim	Orange (peeled fruit)	MSPE	HPLC–MS/MS	0.03 μg/mL	0.10 μg/mL	1.5–4.7	(Farooq et al., 2020)
33	Methamidophos, parathion, phoxim	Orange (juice)	MSPE	HPLC–DAD	0.00006–0.00013 μg/mL	0.00021–0.00044 μg/mL	2.7–4.5	(Du et al., 2019)
34	10 multiclass pesticides	Orange (juice)	DLLME	HPLC–MS/MS	0.000001–0.0001 μg/g	0.000003–0.0003 μg/g	2.5–6.7	(Xue et al., 2019)
35	Chlorpyrifos, triazophos	Orange (whole fruit)	SPE	HPLC–UV	0.0001–0.0003 μg/g	0.20–0.51 μg/g	0.4–9.0	(Wei et al., 2019)
36	Thiabendazole, carbendazim, fuberidazole	Orange (pulp)	SPE	HPLC–FD	0.00003–0.00968 μg/mL	0.00012–0.03236 μg/mL	<8	(Liang et al., 2019)
37	20 Organophosphorus Pesticides (OPPs)	Orange (whole fruit)	MSPE	HPLC–MS/MS	0.000002–0.000063 μg/g	0.001 μg/g	<12.3	(Lin et al., 2020)
38	5 benzimidazoles	Lemon (juice)	MSPE	HPLC–DAD	0.0025–0.0029 μg/mL	0.0088–0.0097 μg/mL	<8.6	(Li et al., 2020)
									39	5 fungicides	Orange (whole fruit)	QuEChERS	HPLC–MS/MS	0.00067–0.00125 μg/g	0.00224–0.00415 μg/g	<7	(Fares et al., 2021)
40	7 fungicides	Orange (juice)	MSPE	HPLC–MS/MS	0.00052–0.00183 μg/mL	–	1.2–2.8	(Liu et al., 2021b)
41	287 multiclass pesticide residues	Citrus (whole fruits)	QuEChERS	HPLC–MS/MS	–	0.001–0.01 μg/g	<20	(Yuan et al., 2024)
42	Thiabendazole	Food samples (solid/liquid)	Methanol extraction	HPLC–DAD	0.009–0.017 μg/mL	0.028–0.052 μg/mL	<3.1	(Choi et al., 2024)
43	Forchlorfenuron, paclobutrazol, uniconazole	Citrus (whole fruits)	SPE	HPLC–MS/MS	0.00009–0.00017 μg/g	0.00029–0.00056 μg/g	1.5–6.3	(Zhang et al., 2023)
44	Albendazole	Citrus (whole fruits)	ACN/acetic acid extraction	HPLC–MS/MS	0.000001–0.01354 μg/mL	0.000003–0.04513 μg/mL	<2.1	(Yang et al., 2023)
45	355 multiclass pesticide residues	Lemon (fruit and juice)	QuEChERS	GC, HPLC–MS/MS	–	0.01 μg/g	<20	(Aslantas et al., 2023)
46	Bifenthrin	Kumquat (whole fruit)	LLE	HPLC–UV	0.003 μg/g	0.01 μg/g	NR	(Chen et al., 2023)
47	Imazalil	Orange (juice)	DLLME, SPE	HPLC-Chiral detection	0.54–0.94 μg/mL	1.80-3.18 μg/mL	4.97	(Ruiz-Rodríguez et al., 2015)
48	Abamectin, spinosad, imidacloprid, difenoconazole	Citrus (whole fruits)	Ethyl acetate extraction	HPLC–MS/MS	–	0.01–0.05 μg/g	<16	(Besil et al., 2019)
49	16 multiclass pesticide residues	Lemon (whole fruit, juice, essential oil)	QuEChERS	GC, HPLC–MS/MS	–	0.01–0.10 μg/mL	<15	(Besil et al., 2019)

LOD, limit of detection; RSD, relative standard deviation; GC-MS/MS, gas chromatography-tandem mass spectrometry; SPE, solid-phase extraction; DLLME, dispersive liquid–liquid microextraction; QuEChERS, Quick, Easy, Cheap, Effective, Rugged, and Safe; GC, gas chromatography; ECD, electron capture detection; FPD, flame photometric detector; MSPE, magnetic solid-phase extraction; SALLE, salting-out assisted liquid–liquid extraction.

#### 4.3.3 Spectroscopy-based non-invasive detection

Spectroscopy-based non-invasive detection of pesticides, such as Raman spectroscopy, is a non-destructive analysis. Two-dimensional Raman correlation spectroscopy enhances spectral resolution and identifies specific pesticide fingerprints, such as chlorothalonil, by focusing on key fingerprint regions. The combination of Raman spectroscopy with principal component analysis (PCA) and support vector machines (SVMs) has shown perfect classification accuracy in detecting chlorothalonil residues in vegetables (Ndung’u et al., 2024a). Surface-enhanced Raman spectroscopy (SERS) is highlighted for its ability to detect pesticides like endosulfan at trace levels using colloidal nanoparticles and aggregating agents. This method is portable and can be applied on-site (Fǎlǎmaş et al., 2024a). SERS also benefits from flexible substrates, such as fluorinated polyimide films, which allow for rapid detection on irregular surfaces (Wei et al., 2023). The use of convolutional neural networks (CNNs) in conjunction with Raman spectroscopy data has improved the classification accuracy of pesticide detection, achieving up to 89.33% accuracy in identifying pesticide compositions (Kuo et al., 2023). Another study demonstrated a CNN model achieving 100% classification accuracy for mixed pesticide detection, underscoring the potential of machine learning in enhancing spectroscopic analysis (Wang et al., 2023a). Advanced statistical models, such as self-modeling curve resolution and multivariate curve resolution, are employed to interpret complex Raman signals. These models are crucial for analyzing multicomponent mixtures and overcoming challenges related to overlapping spectral bands (Sharma et al., 2024). Integration with quantum chemical computation, such as combining SERS with density functional theory calculations, provides structural insights and enhances the identification of compound-specific bands, facilitating the detection of pesticides like paraquat and thiram (Hermsen et al., 2024). Despite the promise of SERS (**Table 6**), reproducibility remains a significant challenge due to variations in substrates, solvents, and equipment. Addressing these issues requires standardized protocols and improved substrate designs (Averkiev et al., 2024b). Another spectroscopy-based method is using volume holography transmission (VHT) gratings. It also offers non-invasive detection of pesticide residues (Wang et al., 2024b).

#### 4.3.4 Fluorescence spectroscopy techniques

Fluorescence spectroscopy involves the excitation of molecules by light, leading to the emission of light at a different wavelength. This property is exploited to detect pesticide residues by measuring the fluorescence intensity, which correlates with the concentration of the pesticide (Ji et al., 2021; Cai and Bian, 2022). Techniques such as aggregation-induced emission (AIE) and the use of nanomaterials like metal nanoparticles and quantum dots are already well developed (Marimuthu et al., 2024; Zha et al., 2024). The use of algorithms such as SVMs, partial least squares regression (PLSR), and back-propagation neural networks (BPNNs) has significantly improved the accuracy of pesticide detection. These models help in distinguishing between different pesticides and quantifying their concentrations even in complex matrices (Bian et al., 2020; Ji et al., 2021; Gao et al., 2025). Data preprocessing methods like convolutional smoothing, standard normal variable transformation, and multiplicative scatter correction are employed to optimize spectral data, enhancing the reliability of the detection models (Gao et al., 2025). Fluorescence spectroscopy has been successfully applied to detect pesticide residues in various samples, including tomato leaves, water, and soil. For instance, the detection of benzyl-pyrazolyl esters on tomato leaves demonstrated high accuracy and reliability, with models achieving R^2^ values close to 1 (Jan et al., 2023; Gao et al., 2025). The challenge of detecting multiple pesticides with overlapping fluorescence spectra has been addressed using multiple PLS models and neural network algorithms, allowing for the simultaneous analysis of several pesticides in a single sample (Bian et al., 2020; Ji et al., 2020).

#### 4.3.5 Support vector machines for qualitative analysis

A non-standard-substance pesticide residue qualitative analysis method using SVMs transforms the problem into a classification task. This method does not require chemical standard substances, making it efficient for qualitative analysis (Iskandar et al., 2021). This is used in combination with other spectroscopic techniques. Raman spectroscopy, combined with SVM, has been used to detect chlorothalonil pesticide residues in vegetables (Ndung’u et al., 2024a). A modified SVM-assisted metabolomics approach has been developed for non-targeted screening of multiclass pesticides and veterinary drugs in maize. This method significantly improves the screening accuracy compared to metabolomics alone, identifying 120 out of 124 contaminants (Xue et al., 2024).

#### 4.3.6 Biosensors

Biosensors have emerged as a promising technology for the detection of pesticide residues. These biosensors utilize biological recognition elements, such as an enzyme, antibody, or DNA, to detect specific substances, providing a cheap, portable, and real-time solution (Gyanjyoti et al., 2022).

##### 4.3.6.1 Enzyme-based and paper-based biosensors

Paper-based biosensors are useful in field applications due to their ease of use and rapid response time. A notable example is the colorimetric paper-based biosensor that uses acetylcholinesterase (AChE) immobilized in sol–gel matrices, optimized using response surface methodology (RSM) for enhanced stability and sensitivity (Wijayanti et al., 2024).

##### 4.3.6.2 Aptasensors

Aptasensors utilize aptamers, which are short DNA or RNA molecules, as recognition elements for pesticide detection. These sensors are known for their high selectivity and sensitivity, as well as their ability to be easily modified for specific applications (Chaskar et al., 2024). Optical aptamer sensors, including fluorescence and colorimetric methods, have been developed for rapid and accurate detection of various pesticides, offering advantages such as quick response times and real-time monitoring capabilities (Guo et al., 2024).

##### 4.3.6.3 Lateral flow test strip biosensors

Optical lateral flow test strip (LFTS) biosensors combine the high sensitivity of optical monitoring with the simplicity and portability of LFTS assays. These biosensors have been developed for single-target and multiplexed detection of pesticides, utilizing colorimetric, fluorescent, and chemiluminescent techniques (Zhang et al., 2021).

#### 4.3.7 Electroanalytical techniques

Electroanalytical techniques have emerged as a vital tool for the detection of pesticide residues due to their high sensitivity, selectivity, and cost-effectiveness. These methods are particularly advantageous for on-site analysis (Souza et al., 2021). Electrochemical biosensors use different electrode materials such as metal–organic frameworks and carbon nanomaterials (Ghanbari et al., 2024; Abedeen et al., 2024). Electrochemical sensors include voltammetry, amperometry, and potentiometry. These sensors operate by converting chemical information into an electrical signal, which is then measured and analyzed. The response of these sensors is largely determined by the electrode materials and the specific electrochemical techniques employed (Kul, 2023; Guo et al., 2024). Advances in electrode materials, such as bimetallic nanoparticles, metal–organic frameworks, and carbon-based materials, have significantly improved the performance of electrochemical sensors. These materials enhance the catalytic activity and facilitate the effective conversion of analyte interactions into electrical signals (Bimetallic Nanomaterials-Based Electroanalytical Methods for Detection of Pesticide Residues, n.d.; Abedeen et al., 2024). Electrochemical sensors have been effectively used to detect organophosphorus pesticides, such as dichlorvos, with high selectivity and low detection limits. The use of composite sensors, like ZrO2@PDA, has demonstrated excellent stability and anti-interference capabilities (Luo et al., 2024). The detection of neonicotinoid pesticides, such as nitenpyram and dinotefuran, has been achieved using voltammetric methods (Kul, 2023; Guo et al., 2024). The use of dental amalgam electrodes for the voltammetric determination of triazine-based pesticides in natural waters has shown promising results. This method provides detection limits below regulatory thresholds (Martins and Souza, 2023).

##### 4.3.7.1 Enzyme-free electrochemical sensors

Enzyme-free electrochemical sensors overcome the limitations of enzyme-based systems, such as stability and cost. These sensors use novel nanoscale materials to enhance detection capabilities (Abedeen et al., 2024).

#### 4.3.8 Fluorescence-based sensors

Nanomaterial-based fluorescence sensors, such as those using metal nanoparticles, carbon dots, and quantum dots, have been developed to detect pesticides like organophosphates and carbamates (Zha et al., 2024). These sensors utilize mechanisms like Förster resonance energy transfer (FRET) and photoinduced electron transfer (PET) to enhance detection sensitivity (Marimuthu et al., 2024).

##### 4.3.8.1 Optical aptamer sensors

Optical aptamer sensors leverage the selectivity and sensitivity of nucleic acid aptamers to interact specifically with target pesticides. These sensors employ fluorescence, colorimetric, and chemiluminescence methods to provide an indication (Guo et al., 2024).

#### 4.3.9 Novel optical sensors

Innovative optical sensors, such as those based on enzyme-free systems, have been designed for the selective and sensitive detection of specific pesticides like glyphosate and malathion. These sensors provide rapid color change and fluorescence response, making them suitable for real sample analysis (Aydin et al., 2022). In a recent study, two novel optical sensors were developed using Fe(III) and Eu(III) Salophen complexes for detecting the organophosphorus pesticide monocrotophos. The Fe(III) Salophen complex forms a supramolecule with monocrotophos, resulting in a strong resonance light scattering signal, with a detection limit of 30 nM and a linear range of 0.1–1.1 μM.^1^ The Eu(III) Salophen complex, combined with 5-aminofluorescein derivatives, forms a sandwich-type supramolecule, with a detection limit of 0.4 μM and a linear range of 1.3–7.0 μM (Li et al., 2023). The integration of advanced materials and techniques, such as nanomaterials and photochemical processes, continues to enhance the performance of these sensors (Huang et al., 2018; Lehotay and Cook, 2015). However, further research is needed to address issues related to sensor stability, cost-effectiveness, and practical application in diverse environmental and food matrices.

## 5 Conclusion and future aspects

The widespread use of pesticides in modern agriculture, while crucial for crop protection, poses significant health and environmental challenges due to the persistence of residues in fruits and vegetables. Numerous studies have confirmed that pesticide levels often exceed MRLs, particularly in developing countries where regulatory oversight may be weaker. This underscores the urgent need for the routine monitoring and global harmonization of residue standards. Analytical advancements especially in extraction and detection techniques such as QuEChERS, GC–MS/MS, LC–MS/MS, and biosensors have significantly improved the sensitivity and specificity of pesticide residue analysis. Emerging green chemistry approaches lead to environmental safety, and AI-driven platforms promise further refinement of detection methods. However, the complexity of food matrices and the diversity of pesticide chemistries demand continuous innovation in both sample preparation and analytical strategies. Strengthening global regulatory frameworks and investing in analytical infrastructure are essential to ensuring food safety and protecting public health in the face of persistent pesticide contamination. As global concerns over pesticide residues in food intensify, future research must focus on developing more sustainable, sensitive, and rapid analytical methodologies. The integration of green extraction techniques, such as NADESs and enzyme-assisted extractions, offers promising eco-friendly alternatives to conventional solvent-intensive protocols. However, the regulatory acceptance of such techniques is critical for their translation from laboratory innovation to routine monitoring systems. Advancements in miniaturized and portable devices, including lab-on-chip platforms and paper-based biosensors, could revolutionize in-field detection by enabling real-time, on-site monitoring. Furthermore, the application of machine learning and artificial intelligence holds immense potential for automating data interpretation, optimizing method parameters, and enhancing predictive modeling of residue behavior in various matrices. Interdisciplinary collaboration among chemists, agronomists, data scientists, and policymakers is essential to develop harmonized regulatory frameworks with standardized global protocols and to support the global implementation of advanced residue monitoring systems. Embracing these innovations will not only improve analytical efficiency but also promote comparability across laboratories, ensuring long-term food safety and environmental sustainability. Plant metabolite profiling and metabolomics are set to revolutionize pesticide residue analysis by enabling more comprehensive, sensitive, and mechanistic assessments of pesticide impacts and residues in plants. The integration of high-throughput, high-resolution techniques (e.g., GC–MS, LC–MS/MS, Ultra-High-Performance Liquid Chromatography (UHPLC), Matrix-Assisted Laser Desorption Ionization (MALDI)–TOF MS, and NMR) is expanding the range and sensitivity of detectable metabolites, allowing for more detailed profiling of both parent pesticides and their transformation products. Next-generation mass spectrometry and bioinformatics are enabling untargeted, large-scale metabolite inventories, supporting hypothesis-driven research and regulatory applications. Meanwhile, the integration of omics technologies with biosensor platforms provides an opportunity to combine mechanistic insights with portable detection, enabling real-time evaluation of pesticide impacts on food safety. Modeling approaches now incorporate the bioconcentration of both parent pesticides and their metabolites, improving risk assessment accuracy and highlighting the need for databases on plant-specific metabolic rates. Combining metabolomics with genomics, transcriptomics, and proteomics (multi-omics) enhances the ability to link metabolic changes to genetic and environmental factors, supporting the development of stress-tolerant crops and improved food safety.
